# Effects of Square-Stepping Exercise on Motor and Cognitive Skills in Autism Spectrum Disorder Children and Adolescents: A Study Protocol

**DOI:** 10.3390/healthcare10030450

**Published:** 2022-02-28

**Authors:** Sabina Barrios-Fernández, Jorge Carlos-Vivas, Laura Muñoz-Bermejo, María Mendoza-Muñoz, Maria Dolores Apolo-Arenas, Andrés García-Gómez, Margarita Gozalo, José Carmelo Adsuar

**Affiliations:** 1 Social Impact and Innovation in Health (InHEALTH) Research Group, Faculty of Sport Sciences, University of Extremadura, 10003 Cáceres, Spain; sabinabarrios@unex.es; 2 Promoting a Healthy Society Research Group (PHeSO), Faculty of Sport Sciences, University of Extremadura, 10003 Cáceres, Spain; mamendozam@unex.es (M.M.-M.); jadssal@unex.es (J.C.A.); 3 Department of Medical and Surgical Therapeutics, Medicine and Health Sciences College, University of Extremadura, 06006 Badajoz, Spain; mdapolo@unex.es; 4 Occupational Stress, Psychopathologies and Emotional Well-Being (GRESPE) Research Group, University of Extremadura, 06006 Badajoz, Spain; agarcil9@unex.es; 5 Psychology and Anthropology Department, University of Extremadura, 10003 Cáceres, Spain; mgozalo@unex.es

**Keywords:** square-stepping exercise, motor skills, balance, cognitive skills, sensory processing, sensory integration, autism spectrum disorders

## Abstract

Individuals with autism spectrum disorder (ASD) diagnoses present not only cognitive, emotional, communicative, and social challenges but also movement issues that affect their everyday activities, learning, and leisure. The use of the square-stepping exercise (SSE), a motor program initially created to strengthen the lower limbs of older adults, is spreading because of its advantages (e.g., balance and lower limb strength improvements). A study protocol to assess the SSE effects on motor, sensory, and cognitive skills in Spanish children and adolescents between 6 and 12 years old with ASD diagnoses is presented. A randomised clinical will be performed, recruiting 52 children and adolescents with ASD who will be distributed into two groups: an experimental (*n* = 26) and a control (*n* = 26) group. The SSE sessions will be held for 9 weeks (two times per week). The main variable will be balance, which will be measured with the Movement Assessment Battery for Children 2 (MABC2), and secondary outcomes will include sensory processing, attention, and executive functions. Assessments will be carried out before and at the end of the program implementation, including an additional follow up one month later. If this program obtains positive results, it should be implemented in different settings (schools, clinics, associations, etc.) to improve the quality of movement and development in children and adolescents with ASD, as it is an easy-to-use and structured tool.

## 1. Introduction

### 1.1. Autism Spectrum Disorders

Conceptualising autism spectrum disorders (ASDs) is a complex task, as their definition has evolved over time [1,2]. It must be remembered that “when you know a person with ASD, you know a person with ASD”, which means that each person is different because they not only have different levels of severity but also different strengths, personalities, environments and settings, and support [3,4]. Thus, ASDs are classified within the group of neurodevelopmental disorders, which also includes intellectual disabilities, communication disorders, attention-deficit/hyperactivity disorder, specific learning disorders, motor disorders, and other neurodevelopmental disorders [5]. ASD is characterised by persistent deficits in social communication and interaction and by restricted, repetitive patterns of behaviours, interests, or activities with clinically significant impairment in their daily functioning and participation [5,6]. When an individual is diagnosed with ASD, it should be specified whether there are comorbidities of intellectual or language impairment, other neurodevelopmental disorders, or medical or genetic conditions. Thus, the severity of the disorder is classified into three levels: 1st level, “supports are required”; 2nd level, “substantial supports are required”; and 3rd level, “very substantial supports are required” [5,7].

People with ASD cope with cognitive, emotional, communicative, and social challenges, including attention [8], working memory, planning, inhibition, impulsivity control or problem-solving [9,10], challenges in self-control and self-regulation [11,12], theory of mind [13,14], issues with intersubjectivity and joint attention [15,16], other challenges related to comprehension and social use of language [17,18], social skills and competence [19,20] in interpreting contexts [21,22], and sensory integration issues [23,24,25], among others.

### 1.2. Motor Impairments in Autism Spectrum Disorder

As mentioned, the main symptoms of ASD are focused on those described in diagnostic manuals [5,26]. However, for some time now, motor performance has been the subject of different studies because motor difficulties are observed both in fine [27,28] and gross motor skills and coordination [29], showing difficulties or delays in acquiring motor milestones or fundamental movement skills [30,31]. Children and adolescents with ASD have issues using the sensory inputs necessary for adequate postural control [32], which is essential for standing, walking, and performing activities of daily living [33]. Balance performance has been also studied. Deficits in sensory integration are well-documented in ASD, with implications of producing purposeful and well-adjusted movement, as the brain requires proper integration of the information from both the internal and external environment to produce appropriate motor responses to the presented challenges [23,34]. People with ASD present difficulties while processing the sensory information necessary for postural control, which results in increased susceptibility to postural sway under visual stimuli in comparison with somatosensory stimuli [32,35]. Regarding gait, they perform longer stance phases and shorter steps, using a greater base of support, greater hip flexion, less knee extension, and an altered contact pattern. Differences in gait cadence and hip and ankle kinetics have also been found, with reduced plantar flexor movements and increased dorsiflexion angle [36,37]. The reasons for these abnormalities include the presence of hypotonia, difficulties in sensory integration and motor planning [23,38,39], and dysfunction of the basal ganglia or cerebellum [36,37,40,41]. Hypotonia is so closely linked to ASD that some studies even indicate that it may be a key element for the early detection of ASD [42]. Low muscle tone may be an early marker of ASD, with a more pronounced influence in younger children. Hypotonia may speed up the age of diagnosis by one and a half years on average in boys and one year in girls [43]. Moreover, hypotonia in children with ASD presents in 15–67% of cases; is associated with other motor abnormalities, such as a higher frequency of motor stereotypies and later onset of independent walking; and could be an early marker of increased severity of autism symptomatology, leading to a lower quality of life in young children with ASD and their families. [44]. Therefore, it seems important to provide children with autism with opportunities for movement to enhance their motor, cognitive, and social development [45,46].

### 1.3. Square Stepping Exercise

The square-stepping exercise (SSE) comprises the performance of movement patterns, including forward, backward, lateral, and oblique stepping, becoming progressively more complex [47]. The SSE was designed to improve reaction time during the step as well as restore balance after a stumble, as it involves the activation of the agonist and antagonist muscles of the lower limbs [47,48]. This program is carried out on a fine mat of 200 × 100 cm divided into 40 squares of 25 × 25 cm, which is adapted to the individual’s characteristics. Participants start with movement patterns such as walking, and little by little, they make more complex patterns that require multidirectional movements [49]. The SSE includes close to 200 diverse movement patterns, classified by difficulty into three general levels: beginner (including two sub-levels), intermediate (with three sub-levels), and advanced (with three sub-levels), but new or adapted pattern proposals could be added to improve the attention and motivation of the participants (e.g., including other body parts, colours, objects, etc.). Therefore, it can be considered a low-cost modality of exergaming, which can be developed indoors or outdoors, providing an innovative and playful sensorimotor intervention tool to develop skills in children and adolescents with ASD.

Using the SSE, motor and cognitive improvements have been found in functionality, balance, lower limb strength, flexibility and agility, attention, and memory in older adults [50,51]. It has shown its effectiveness in preventing and reducing falls [52]. It has been applied to diseases such as type 2 diabetes mellitus [53] and multiple sclerosis [54]. However, we are only aware of one study performed with pre-schooler children; the study aimed to determine whether SSE improved children’s cognitive skills by recruiting a sample of 28 pre-schoolers, with 18 of them assigned to an experimental group that performed SSE three times a week for eight weeks. The data revealed statistically significant improvements between the two groups [55].

### 1.4. Aim

The SSE is an intervention that is rarely used in children [55,56], although it offers numerous advantages, such as being low cost, portable, adaptable, and modifiable, with different levels of difficulty and options to include different kinds of aids. Thus, we aim to analyse the effects of an SSE training program in motor (balance), sensory processing (sensory patterns, systems, and adaptive behaviour) and cognitive (attention and executive function) skills in children and adolescents with ASD.

We hypothesise that the SSE programme will lead to sensorimotor improvements in children with ASD because of improvements in sensory integration and balance and postural control. Moreover, because of the large component of planning, sequencing, change of criteria, and monitoring, we also expect positive results in the enhancement of attention and executive functions.

## 2. Materials and Methods

### 2.1. Study Design

A randomised controlled trial will be conducted with a 1:1 allocation ratio to experimental and control groups, following the Consolidated Standards of Reporting Trials (CONSORT) statement [57].

### 2.2. Ethical Approval

This trial was approved by the Bioethics Committee of the University of Extremadura (162/2021). Moreover, it was registered in the Clinical Trials Registry (request number: 383354) provided by the Australian New Zealand Clinical Trial Registry (https://www.anzctr.org.au/) (accessed on 2 August 2021).

### 2.3. Sample Size

Assuming an alpha risk of 0.05 and a beta risk of 0.2 in a bilateral contrast, and assuming an effect size of 0.40 (large), 52 subjects (26 individuals in both the experimental and control groups) are required to achieve an 80% statistical power.

### 2.4. Eligibility Criteria

Participants will meet the following criteria: (a) age between 6 and 12 years; (b) a clinical ASD diagnosis under The Diagnostic and Statistical Manual of Mental Disorders, Fifth Edition [5]; (c) ability to understand and follow simple commands; (d) no inconvenience that prevents physical activity; (e) children and adolescents’ voluntariness to take part in the study; and (f) provide signed informed consent form by the legal guardians. The experimental group individuals should complete at least 80% of the sessions and all the evaluations to be considered in the analysis.

### 2.5. Randomisation and Blinding

Participants will be randomly assigned to the experimental (SSE) or control (usual care) group using The Research Randomizer (http://www.randomizer.org) (accessed on 8 July 2021) [58], with a computer-generated randomisation sequence (1:1). A member of the team not involved in this trial will handle this task, protecting the assignment in a password-protected computer file. Participants will be aware of which group they have been assigned to, but the researchers who will carry out the measurements and analyses will not.

### 2.6. Instruments

Movement Assessment Battery for Children 2 (MACB-2): This battery consists of (1) a standardised test composed of eight tests that assess three dimensions of movement (manual dexterity, aiming and catching, and balance), with different tests divided into three age ranges (3–6, 7–10, and 11–16 years); and (2) a behavioural observational checklist related to motor activities of daily living [59], one of the most frequently used instruments for coordination motor competency in children between 3 and 16 years old [60]. The psychometric properties are adequate, with reliability values above 0.70 on all scales, an intraclass correlation coefficient of 0.97, minimal important difference values from 2.36 to 2.50, and consistent validity when differentiating children with and without motor dysfunctions [59,61]. The balance section, which assesses static and dynamic balance through different tests for the different age ranges (Table 1), will be used for this study.

The Behavioural Observation Checklist: This checklist, included in the MACB-2 [59], allows the observation of specific motor skills that occur in everyday environments. It can be administered by parents, teachers, or professionals who know the child well. It can be used for children between 5 and 12 years of age and is composed of three sections. Section A is entitled “Moving in a static and/or predictable environment.” It consists of 15 items and is scored on a Likert scale from 0 to 3, where 0 means “very well” and 3 means “with great difficulty”. There is a response option for unobserved behaviours. Section B, “Moving in a dynamic and/or unpredictable environment”, is composed of 15 items and is scored the same as section A. Section C, “Non-motor factors that may affect movement”, consists of 13 dichotomous responses items (yes or no). It has a high internal consistency (α ≥ 0.90) including a wide repertoire of motor skills that children perform in their daily lives. Its concurrent validity is moderate. Although its sensitivity is limited, it has good specificity [60].

Sensory Profile 2, Short Form (SSP2): This questionnaire evaluates sensory processing by providing information on different sensory patterns (seeking, avoiding, sensitivity, and registration), through the sensory systems (auditory, visual, tactile, and oral processing), obtaining information about adaptive behaviours (socioemotional and attention) in children between 3 years and 14 years and 11 months. The Spanish version consists of three versions: the children, scholar, and brief versions. The reliability values are above 0.70 on all factors and versions [62]. In this study, the children’s version will be used. Its internal consistency is between adequate (α = 0.72) and excellent (α = 0.90), and its test–retest reliability coefficients range between 0.93 and 0.97 [62].

For the evaluation of attention and executive function, the following are proposed:

- Test on Perception of Differences (FACES), in the Spanish version [63], assesses perceptual and attentional skills using 60 graphic items consisting of schematic drawings of faces with elementary strokes. It consists of choosing which of the three faces that make up each element is different from the other two. It is a very brief test (three minutes) that can be applied to children and adolescents between 6 and 18 years old. The overall Cronbach’s alpha was 0.91, with a range of values being between 0.82 and 0.92. In the case of validity, it obtained moderate correlations with other intelligence and reasoning tests [63].
- Short Version of the Executive Functioning Questionnaire (EFECO-S) [64], which is composed of 20 items divided into five factors: emotional self-control, initiative and planning, working memory, inhibition, and organisation, which allow screening for difficulties in daily executive functioning. It can be completed by both parents and relatives and is valid for children and adolescents between 6 and 13 years of age. The Confirmatory Factor Analysis provided 5-factor solutions with good and excellent goodness of fit indices. The reliability, established through ordinal alpha, was high, and the magnitude of the correlation between the original version of the EFECO questionnaire and the short version was also high, which is an important support for validity [64].
- Rings and Interference Tests from the Neuropsychological Assessment of Executive Functions in Children (ENFEN) [65]. The ring test consists of the reproduction, on a board with three vertical axes, of a model presented on a sheet (towers task); whereas the interference test consists of a list of 39 words representing colour names (red, green, yellow, and blue) printed in ink of a different colour. The task consists of naming the ink colour of the word, inhibiting the written word (Stroop paradigm). Its results are validated for use in children and adolescents between 6 and 12 years of age. The alpha value was 0.76, with a 95% confidence interval between 0.73 and 0.78 [65].

### 2.7. Intervention

Experimental group: Participants will perform the SSE intervention program twice a week for 9 weeks. The instructors will receive initial training on ASD (characteristics, strengths, motivation, and disruptive behaviour management techniques) and will be familiarised with the participants before starting the intervention. The training sessions will last approximately 30 min, and their basic structure is shown in Table 2. The first activity will be a general warm-up; then, the participants will learn and execute the selected patterns for that day, and they all should be repeated 5 times. The session will finish with a cool-down.

**Figure 1:**
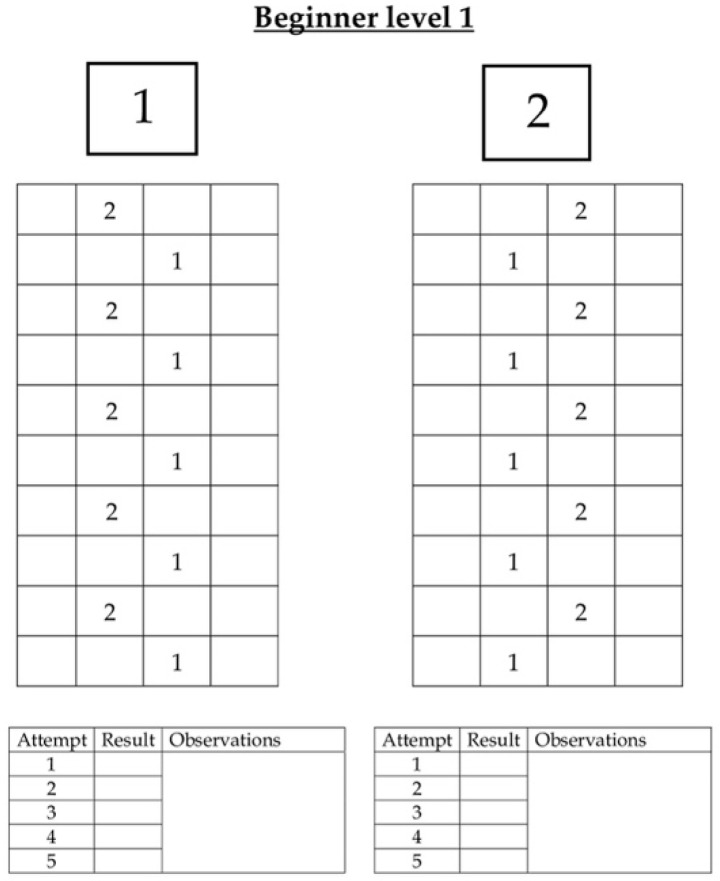
Example of the first two patterns of the initial level. Participants will have to reproduce these patterns 5 times, and the instructors will record the results of each attempt.

Participants will start with movement patterns such as walking, and little by little, they will make more complex patterns that will require multidirectional movements. Participants should not step on the squares’ dividing lines. The instructor will mark every attempt in an individual notebook where the patterns will be registered, providing information on whether they were performed correctly, with errors or with assistance, and qualitative observations can be added. An example can be found in Figure 1.

The SSE has some strengths when used with children and adolescents with ASD: (1) it is simple; the repetitive and easy-to-follow structure provides the anticipation, routine, and comprehension that some children with ASD diagnoses need, promoting errorless teaching that can help participants with self-regulation issues; (2) at the same time, it is flexible, as it offers the possibility to add additional elements, such as songs, colours, balloons, visual aids, and others, which could improve the adherence and pleasure during the training sessions. Table 3 displays the proposed progression of the SSE sessions.

Control group: Participants with ASD will continue with their usual treatments and will only be present for the assessment sessions. An additional control group of age-matched, typically developed children will be added because this intervention is rarely used in children in general.

Both groups will perform evaluations at the beginning of the program, the end of the program, and a follow-up after one month following the end of the training sessions, using the described tools: MACB-2 (balance sections and behavioural checklist), SP2 (child version), the FACES, EFECO-S and Ring and Interference tasks from the ENFEN.

### 2.8. Statistical Analysis

The Statistical Package for the Social Sciences (SPSS, Version 25, IBM SPSS, Armonk, NY, USA) software will be used to perform the analyses.

Data normality and homogeneity will be assessed with the Kolmogorov–Smirnov test and Levene’s test. Descriptive data will be expressed as means and standard deviation (SD) (for normally distributed variables) or medians and interquartile range (for non-normally distributed variables). Then, data will be analysed by intention-to-treat and protocol.

Intention-to-treat analysis. All randomised participants will be considered for the analysis in its group. Missing data will be imputed through multiple imputations. Repeated measures analyses of covariance adjusted by age and baseline outcomes will be used to analyse the intervention effects on the different dependent variables. The effect sizes (95% confidence interval) and differences will be calculated for every variable concerning the time and group × time interaction. The alpha level will be set at *p* < 0.05. The data imputation for sensitivity analyses will be carried out on individuals who present data at baseline and at the end of the trial to prevent estimation bias [66].

Analysis by protocol. The analyses previously described will be carried out, but only for participants who attend at least 80% of the program sessions.

## 3. Discussion

This study will be the first to use an SSE program to improve sensory, motor, and cognitive skills in children and adolescents with ASD diagnoses. To the best of our knowledge, it will be the second in the world carried out in children [55]. We hypothesise that the SSE programme will lead to sensorimotor improvements in children with ASD because of an improvement in sensory integration and balance and postural control. Moreover, because of the high component of planning, sequencing, change of criteria, and monitoring, we also expect positive results in the enhancement of attention and executive functions.

Children and adolescents with ASD present cognitive-behavioural, socio-communicative issues and sensorimotor challenges that cause issues in both fine and motor skills and coordination. The relationship between motor performance and movement quality is closely related to the severity level of the disorder [67,68]. Moreover, motor [35,37,43], sensory [69], cognitive [46], and socio-communicative skills impairments [45,70,71,72] impact the development of their occupations, including activities of daily living, learning, leisure, and social participation in their communities [73,74]. If this program is effective, it should be implemented in schools, clinics, and associations specialising in the education and treatment of ASD individuals because it is an innovative and easy-to-apply program, resulting in a tool for the improvement of the aforementioned skills and the inclusion of these children and adolescents.

In the long term, we also hope to increase the time spent performing physical exercise as a protective factor for their health and quality of life, adapting the activity to their characteristics and considering the barriers. In 2008, Spain ratified the Convention on the Rights of Persons with Disabilities [75], committing itself to carry out policies to improve the social participation of all people in the spheres of daily life, including school and leisure. The right of people with ASD to participate in activities is also included in the Sustainable Development Goals, being directly related to goals (3) Health and well-being, (4) Quality education, and (10) Reduction of inequalities [76]. Therefore, we hope that this project will be a small contribution to fulfilling the rights of people with ASD. One of the most important manifestations of motor skills is the practice of physical activity and sports. Several barriers to physical activity practice in children and adolescents with ASD are reported. Families consider it important for their children with ASD to exercise, but they demand support so that they can participate in these activities, as they are socially highly demanding, occur in changing contexts, and may have rules that are difficult to interpret. All the previous factors, together with their low physical competence and movement difficulties, can cause rejection [77]. Additionally, adults with ASD indicate a lack of motivation, interpersonal difficulties, and problems with sportive activities, including the type of activity, transportation or cost [78]. This limited participation in physical activities causes both preadolescents and adolescents with ASD to have a worse fitness level [79] and a higher body mass index compared with typically developed children and adolescents [80], which causes them to be at risk of suffering from pathologies related to physical inactivity, such as diabetes, hypertension, coronary and cerebrovascular diseases, and overweight/obesity, among others, which increase mortality [81,82].

## 4. Conclusions

The present study will investigate the efficacy of SSE in children and adolescents with ASD for 9 weeks to improve sensory, motor, and cognitive skills. If the efficacy of the program is demonstrated, it could be implemented in centres, entities, and associations specialised in the education and treatment of children and adolescents with ASD diagnoses.

## Figures and Tables

**Table 1:** Tests that compose the balance section according to the age ranges.

Age Ranges	Tests in That Age Range
Age range 1 (4–6 years)	Balance on one leg (best leg/worst leg) Tiptoe walking Jumping on mats
Age range 2 (7–10 years)	Balance on one leg (best leg/worst leg) Forward heel–toe walking Jump on one leg over a line (better leg/another leg)
Age range 3 (11–16 years)	Balancing on two supports Backward heel–toe walking Jump on one leg in zigzag (best leg/worst leg)

**Table 2:** Basic structure of an SSE program session.

**Warm-up** (5 min)
Motor games Joint mobility and stretching
**Main Part** (20 min)
Review of the patterns learnt in the previous session. Learning and realisation of pattern number X. Learning and realisation of pattern number XX *
**Cool-down** (5 min)
Relaxation and comments about the session Personal hygiene

* The intervention will start with beginner level 1 (sub-level 1) patterns and will not move on to the next one until the participant can perform them successfully.

**Table 3:** Planned Progression of SSE intervention proposal.

Week	Frequency (Days a Week)	Session Duration (Minutes)	Steps Per Sequence (Number)	Difficulty (Level)
1	2	30	2	Beginner 1
2	2	30	4	Beginner 2
3	2	30	4	Beginner 2
4	2	30	6	Beginner 2
5	2	30	6	Beginner 2
6	2	30	6	Intermediate 1
7	2	30	8	Intermediate 1
8	2	30	8	Intermediate 1
9	2	30	8	Intermediate 1

## Data Availability

When the programme is carried out, the data will be available upon reasonable request.
